# Genomic loss of EZH2 leads to epigenetic modifications and overexpression of the HOX gene clusters in myelodysplastic syndrome

**DOI:** 10.18632/oncotarget.6992

**Published:** 2016-01-23

**Authors:** Feng Xu, Li Liu, Chun-Kang Chang, Qi He, Ling-Yun Wu, Zheng Zhang, Wen-Hui Shi, Juan Guo, Yang Zhu, You-Shan Zhao, Shu-Cheng Gu, Cheng-Ming Fei, Xiao Li

**Affiliations:** ^1^ Department of Hematology, Shanghai Jiao Tong University Affiliated Sixth People's Hospital, Shanghai, China

**Keywords:** myelodysplastic syndrome, EZH2, H3K27 methylation, overexpression, HOX genes

## Abstract

The role of EZH2 in cancer is complex and may vary depending on cancer type or stage. We examined the effect of altered EZH2 levels on H3K27 methylation, HOX gene expression, and malignant phenotype in myelodysplastic syndrome (MDS) cell lines and an *in vivo* xenograft model. We also studied links between EZH2 expression and prognosis in MDS patients. Patients with high-grade MDS exhibited lower levels of EZH2 expression than those with low-grade MDS. Low EZH2 expression was associated with high percentages of blasts, shorter survival, and increased transformation of MDS into acute myeloid leukemia (AML). MDS patients frequently had reductions in EZH2 copy number. EZH2 knockdown increased tumor growth capacity and reduced H3K27me3 levels in both MDS-derived leukemia cells and in a xenograft model. H3K27me3 levels were reduced and HOX gene cluster expression was increased in MDS patients. EZH2 knockdown also increased HOX gene cluster expression by reducing H3K27me3, and H3K27 demethylating agents increased HOX gene cluster expression in MDS-derived cell lines. These findings suggest genomic loss of EZH2 contributes to overexpression of the HOX gene clusters in MDS through epigenetic modifications.

## INTRODUCTION

The molecular pathogenesis of myelodysplastic syndromes (MDS) remains poorly understood due to their high heterogeneity and complexity of the disease [1]. Recently, however, gene mutations involving epigenetic modifiers have been identified in MDS [2–5]. Further studies of epigenetic modifiers may help clarify the process of tumorigenesis in MDS [6].

Enhancer of zeste homolog 2 (EZH2) is a highly conserved histone methyltransferase that targets lysine 27 of histone H3 (H3K27) [7]. Methylated H3K27, especially trimethylated H3K27, is commonly associated with the silencing of genes involved in cellular development and differentiation [8]. Links between EZH2 and tumorigenesis have been described for various solid cancers [9–13]. Overexpression of EZH2 is frequently detected in cancerous tissues and is correlated with advanced stages of disease and with a poor prognosis. Several studies of hematopoietic system diseases have shown that EZH2 overexpression is involved in lymphoma and leukemia [14–16]. However, it is still debated whether EZH2 acts as an oncogene or a tumor suppressor gene (TSG) in MDS [17–19]. EZH2 mutations were identified in approximately 6% of MDS patients, and these mutations predict poor survival [3, 18, 19]. Nevertheless, most MDS patients do not carry EZH2 mutations, and the role of EZH2 expression in MDS pathogenesis remains unknown.

The homeobox (HOX) gene clusters encode a large family of highly conserved transcription factors that control cell fate during embryogenesis [20]. The expression of HOX gene clusters gradually decreases during hematopoietic cell maturation [21], which suggests that the HOX gene clusters may contribute to maturation defects in MDS clonal cells and pathogenesis. Because HOX gene expression is regulated in part by epigenetic mechanisms, the association between EZH2 and the HOX genes merits further investigation. Here, we examined the role of EZH2 in MDS pathogenesis and its relationship to HOX genes.

## RESULTS

### Patient characteristics

A total of 97 MDS patients, including 57 males and 40 females, were included in this study. Their median age was 58 years (17–85 years). They were classified as RA/RARS (*n* = 15), RCMD/RCMD-RS (*n* = 25), MDS-U (*n* = 8), RAEB-1 (*n* = 23), or RAEB-2 (*n* = 26). Patient characteristics are shown in Supplementary Table 1.

### Decreased EZH2 expression is common in MDS and is associated with poor clinical outcome

**Figure 1:**
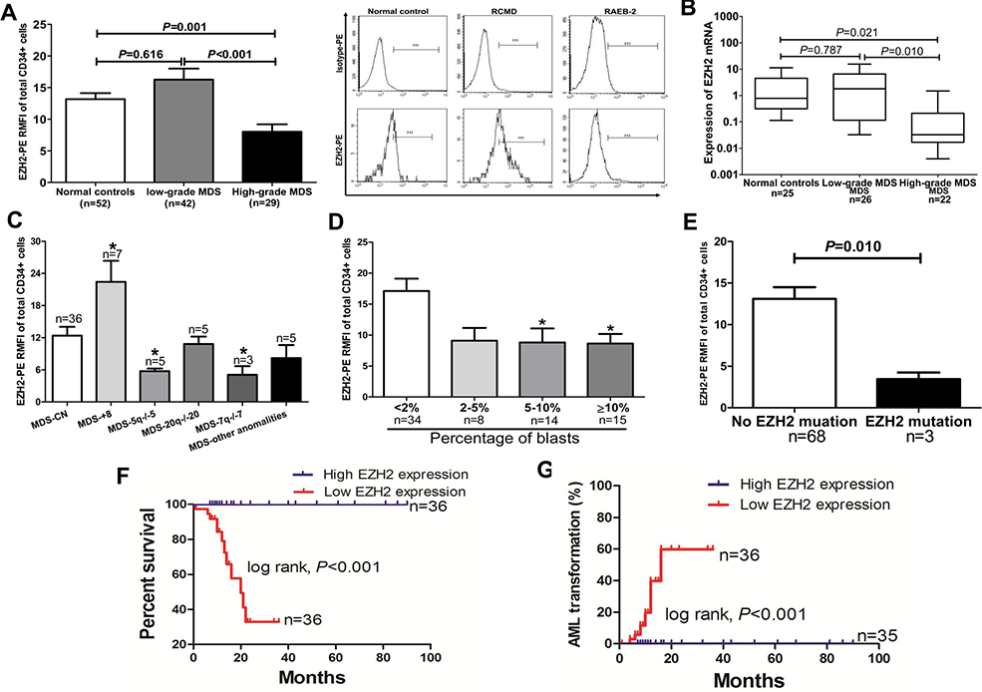
Reduced EZH2 protein levels are common in high-grade MDS and associated with poor clinical outcomes (**A**) FCM analysis showed that patients with high-grade MDS (*n* = 29) had lower EZH2 expression in CD34+ cells than the low-grade MDS group (*n* = 42) and the normal controls (*n* = 52) (*n* = 0.001; *p* < 0.001); A representative graph of RCMD and RAEB-2 in normal controls is shown on the right. (**B**) Reduced EZH2 expression was observed in CD34+ cells from the high-grade MDS group (*n* = 22) compared to the normal controls (*n* = 25) and the low-grade MDS group (*n* = 26) (*n* = 0.021; *n* = 0.010). (**C**) Patients with 7q−/−7, −20/20q−, and 5q−/−5 showed reduced EZH2 expression, whereas those with trisomy 8 had elevated EZH2 expression, compared to patients with normal karyotypes. (**D**) Patients with over 2% of blasts had relatively low EZH2 expression. (**E**) Three cases with EZH2 mutations showed reduced EZH2 expression. (**F**) and (**G**) Patients with low EZH2 expression had shorter overall survival and higher AML transformation (*p* = 0.002; *p* = 0.004). The comparison analysis among the normal controls, the low-grade group, and the high-grade MDS group was performed using ANOVA test. Statistical significance relative to the control group is indicated: *, *p* < 0.05; **, *p* < 0.01; ***, *p* < 0.001 (two-tailed, student *T*-test). Error bars show SEM.

EZH2 protein levels were determined by flow cytometry (FCM) in 71 MDS patients and 52 control individuals. EZH2 expression in CD34+ cells was lower in the high-grade MDS group compared to normal controls and the low-grade MDS group (*p* = 0.001; *p* < 0.001) (Figure 1A). Expression in granulocytes did not differ among the three groups (Supplementary Figure S1A). Lymphocytes and erythroblasts did not express EZH2 (Supplementary Figure S1B and S1C). Quantitative PCR analysis in CD34+ cells showed that EZH2 mRNA levels were also lower in the high-grade MDS group compared to normal controls and the low-grade MDS group (Figure 1B). Individuals with abnormal karyotypes (5q−/−5 and 7q−/−7) show reduced EZH2 expression, while those with +8 karyotypes show higher expression, compared to individuals with normal karyotypes (Figure 1C). Additionally, patients with lower EZH2 expression had a relatively high percentage of blasts (> 2%) (Figure 1D). Three patients with EZH2 mutations (S619F, A651V and R497Q) showed reduced EZH2 expression (Figure 1E). To investigate the influence of EZH2 expression on overall survival and transformation into acute myeloid leukemia (AML), we divided patients into two EZH2 expression groups: low EZH2 expression (RMFI value ≤ median value) and high EZH2 expression (RMFI value > median value). A log-rank test showed that the low EZH2 expression group had shorter overall survival and increased AML transformation compared to the high EZH2 expression groups (*p* = 0.002, Figure 1F; *p* = 0.004, Figure 1G). Finally, Multivariate Cox analysis revealed that the combination of low EZH2 expression together with higher IPSS scores was an independent prognostic factor (HR: 2.473; 95% CI: 1.212–3.845; *p* = 0.014).

### Genomic loss of EZH2 leads to low EZH2 expression

**Figure 2:**
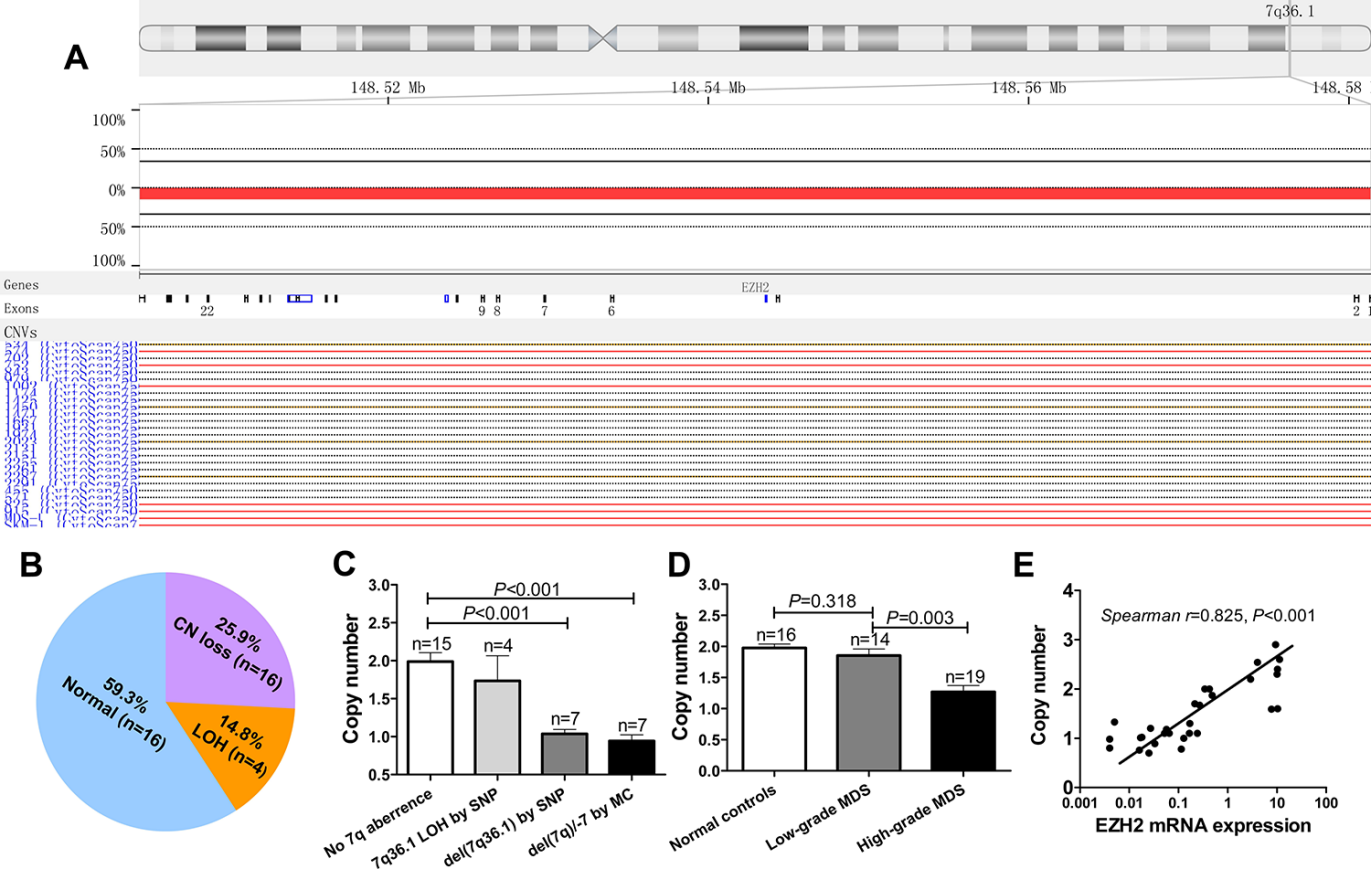
SNP and real-time quantitative PCR reveal lower EZH2 CN in MDS (**A**) and (**B**) SNP arrays were performed in 27 MDS patients with normal karyotypes. CN loss (25.9%) and LOH (14.8%) at the EZH2 locus were frequently observed in MDS patients. (**C**) QRT-PCR analysis based on TaqMan Copy Number Assay confirmed that patients with 7q abnormalities identified by SNP array or MC had lower EZH2 CN (*p* < 0.001; *p* < 0.001). (**D**) Patients with high-grade MDS frequently exhibited CN loss in the EZH2 locus compared to patients with low-grade MDS and the normal controls (*p* = 0.003; *p* < 0.001). (**E**) EZH2 DNA CN was positively correlated with EZH2 mRNA expression (*p* < 0.001). *The comparison analysis was performed using an ANOVA. The correlation analysis was performed using a Spearman analysis*. *Error bars show SEM*.

The EZH2 gene is located at 7q36.1, and 7q−/−7 is one of the most common karyotype abnormalities. As expected, a single nucleotide polymorphism (SNP) microarray showed that a copy number (CN) gain in the chr8 region and CN loss in the chr 7 and chr 5 regions were the most frequent cytogenetic events (Supplementary Figure S2). In 27 patients with normal karyotypes (identified by metaphase cytogenetics), the SNP array identified seven cases (25.9%) with CN loss and four cases (14.8%) with loss of heterogeneity (LOH) at the 7q36.1 locus (Figure 2A and 2B). Quantitative genomic PCR showed that CN loss at the EZH2 locus occurred more frequently in patients with 7q abnormalities, as determined both by SNP array and metaphase cytogenetics (MC), compared to patients without chromosome 7 abnormalities (*p* < 0.001; *p* < 0.001) (Figure 2C). Additionally, CN loss at the EZH2 locus was more common in patients with high-grade MDS than in those with low-grade MDS or normal controls (*p* = 0.003; *p* < 0.001) (Figure 2D). EZH2 DNA CN was positively correlated with EZH2 mRNA expression (Spearman's *r* = 0.825, *p* < 0.001) (Figure 2E). Taken together, these results suggest that genomic loss of EZH2 leads to low EZH2 expression in MDS.

### Knockdown of EZH2 enhances malignant phenotypes in an MDS-derived cell line with reduced H3K27me3 levels

**Figure 3:**
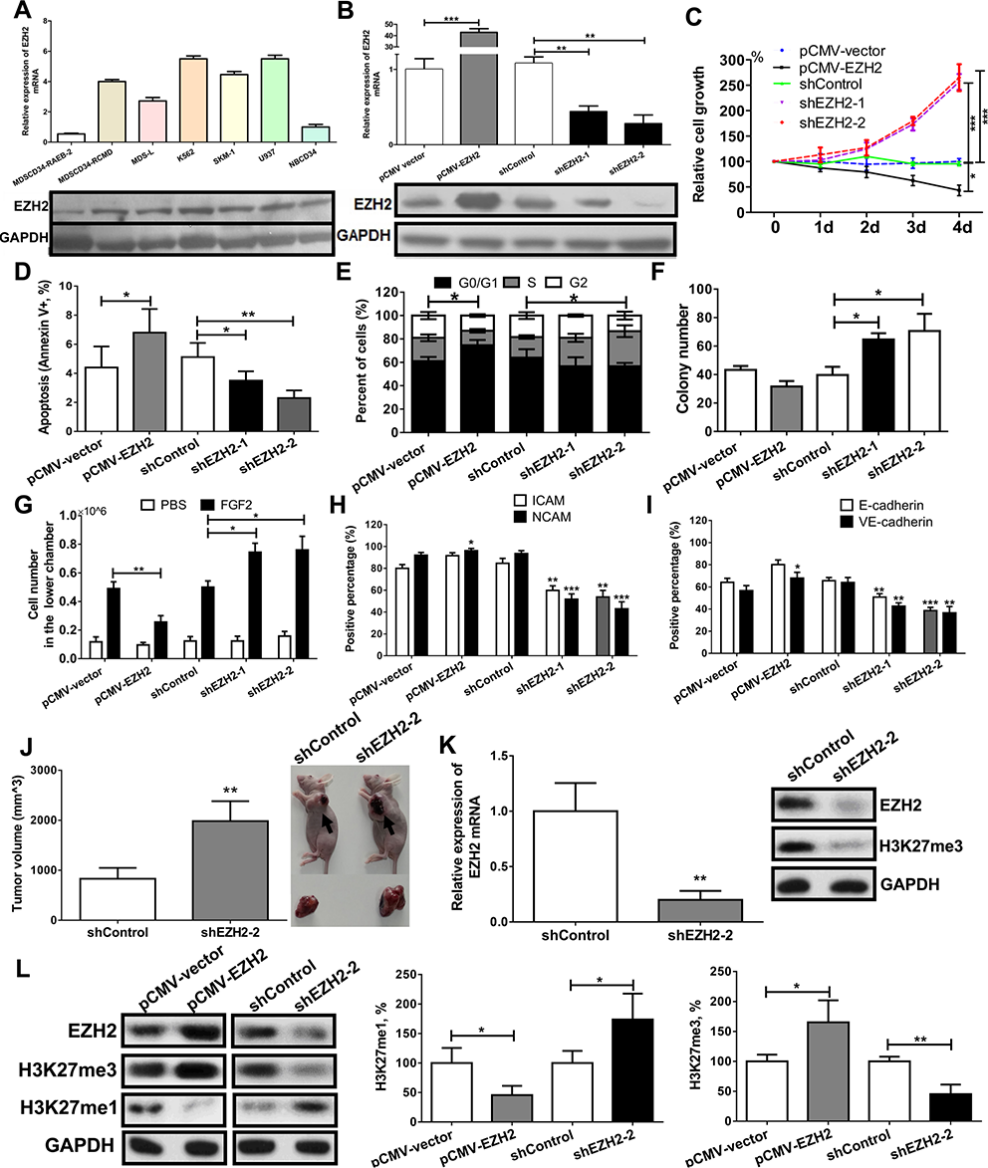
EZH2 knockdown enhances malignant phenotypes in an MDS-derived cell line (**A**) EZH2 mRNA and protein expression was increased in patients with MDS-RCMD and in MDS/leukemia cell lines, but decreased in patients with MDS-RAEB-2. (**B**) SKM-1 cells with stable overexpression and knockdown of EZH2 were obtained through a lentivirus-mediated transfection system. (**C**) EZH2 knockdown increased proliferation, and EZH2 overexpression reduced proliferation. (**D**) EZH2 knockdown reduced cell apoptosis while EZH2 overexpression increased apoptosis. (**E**) EZH2 knockdown induced an incremental increase in the percentage of cells in S phase, while EZH2 overexpression induced arrest in the G0/G1 phase. (**F**) EZH2 knockdown increased colony formation. (**G**) EZH2 knockdown increased cell migration while EZH2 overexpression reduced cell migration. (**H**) and (**I**) EZH2 knockdown decreased the expression of adhesion molecules, whereas EZH2 overexpression increased the expression of adhesion molecules. (**J**) EZH2 knockdown promoted tumor formation in a xenograft model compared to control cells. mRNA and protein analyses identified low EZH2 expression and reduced H3K27me3 levels in xenografts with EZH2 knockdown. (**K**) Western blot analysis showed that EZH2 knockdown reduced H3K27me3 levels and increased H3K27me1 levels, whereas EZH2 overexpression increased H3K27me3 levels and reduced H3K27me1 levels. H3K27 methylation colorimetric analysis confirmed these results. For cell biological experiments, every assay was carried out three or four times. Representative images are shown. Statistical significance relative to the control group is indicated: *p < 0.05; **p < 0.01; ***p < 0.001 (two-tailed, Student's *t*-test). Error bars show SEM.

Expression analysis showed that EZH2 was overexpressed in MDS-L, SKM-1, K562 and U937 cells (Figure 3A). Because they had high EZH2 expression, possess characteristics of MDS clonal cells, and are suitable for lentivirus transfection, we used SKM-1 cells to perform a series of functional experiments.

We constructed SKM-1 cell lines with stable overexpression and knockdown of EZH2 using a lentivirus-mediated transfection system. QRT-PCR and western blot analysis showed an approximately 40-fold increase in EZH2 levels in the overexpression cells and an 80% decrease in EZH2 levels in the knockdown cells (Figure 3B). EZH2 knockdown led to a significant proliferation advantage (Figure 3C). In contrast, EZH2 overexpression increased cell apoptosis and inhibited growth (Figure 3C and 3D). EZH2 knockdown also increased the percentage of cells in S phase, colony formation, and cell migration (promoted by fibroblast growth factor 2) (Figure 3E–3G). In contrast, EZH2 overexpression induced cell cycle arrest in the G1 phase and inhibited cell migration (Figure 3E and 3G).

The adhesion molecules ICAM, NCAM, E-cadherin, and VE-cadherin are crucial for leukemia cell adhesion and migration. EZH2 knockdown reduced the expression of these adhesion markers in SKM-1 cells, while overexpression increased their expression (Figure 3H and 3I). *In vivo* xenograft experiments showed that EZH2 knockdown increased tumor volume by day 20 compared to injection with an shRNA control (Figure 3J). QRT-PCR and western blot analysis confirmed that xenografts with human EZH2 knockdown showed low EZH2 expression and reduced H3K27me3 levels (Figure 3K).

In addition, we analyzed the effect of EZH2 on H3K27 methylation in SKM-1 cells. Western blot analysis showed that EZH2 knockdown reduced H3K27me3 levels and increased H3K27me1 levels; EZH2 overexpression had opposite effects (Figure 3L). Colorimetric analysis of H3K27 methylation confirmed these results.

### Reduced H3K27me3 in the HOX genes cluster is associated with low EZH2 expression in MDS primary CD34+ cells

**Figure 4:**
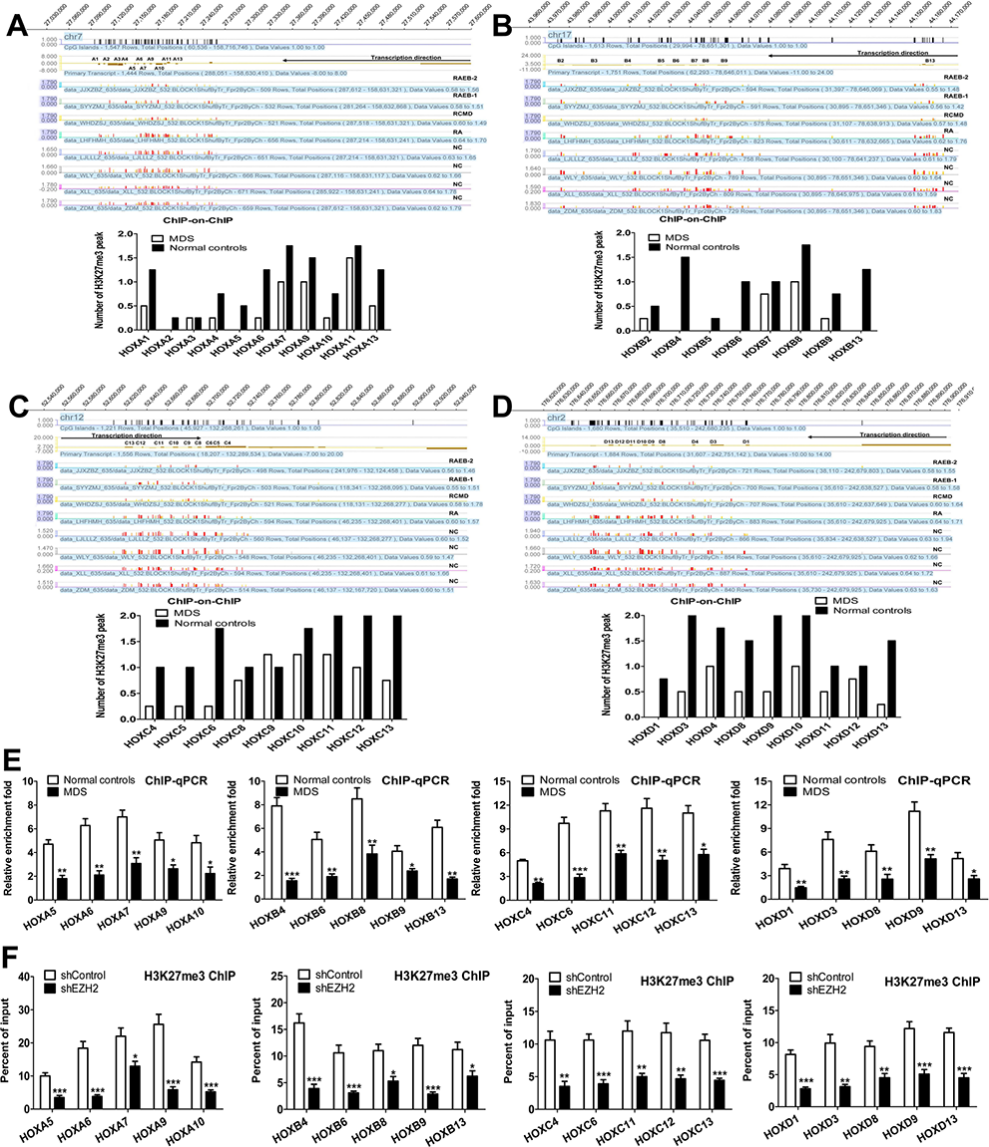
Reduced H3K27me3 in the HOX gene cluster is associated with low EZH2 expression in MDS primary CD34+ cells (**A**) ChIP-on-ChIP analysis revealed widely reduced H3K27me3 levels in the HOXA cluster locus in MDS patients. Reduced H3K27me3 peaks were observed in RAEB-1/2, RCMD, and RA compared to the normal controls. (**B**), (**C**) and (**D**) Similarly, ChIP-on-ChIP analysis revealed widely reduced H3K27me3 levels in HOXB, HOXC, and HOXD cluster loci in MDS patients. (**E**) ChIP-qPCR analysis revealed decreases in H3K27me3 levels at the HOXA, HOXB, HOXC, and HOXD loci in MDS patients. (**F**) ChIP-qPCR showed that EZH2 knockdown reduced H3K27me3 at the HOXA, B, C, and D cluster promoters in SKM-1 cells. Statistical significance relative to the control group is indicated: *p < 0.05; **p < 0.01; ***p < 0.001 (two-tailed, Student's *t*-test). Error bars show SEM.

EZH2 expression was lower in the high-grade MDS group than in the low-grade MDS group. To assess the genome-wide effects of reduced EZH2 on H3K27 methylation, we performed a ChIP-on-ChIP analysis in MDS primary CD34+ cells from eight patients with MDS and eight normal controls. Genome-wide H3K27me3 was reduced in the MDS groups compared to the normal controls (Supplementary Figure S3). As shown in Figure 4, the MDS groups had reduced H3K27me3 in the HOXA (Figure 4A), HOXB (Figure 4B), HOXC (Figure 4C) and HOXD (Figure 4D) cluster loci compared to the normal controls. Furthermore, H3K27me3 was reduced to a greater extent in the high-grade MDS group than in the low-grade MDS group in these loci (Supplementary (Supplementary Figure S4A–S4D). Additional ChIP-qPCR analysis identified a large decrease in H3K27me3 levels in the following HOX loci: HOXA5, A6, A7, A9, and A10; HOXB4, B6, B8, B9, and B13; HOXC4, C6, C11, C12, and C13; and HOXD1, D3, D8, D9, and D13 (Figure 4E).

### EZH2 knockdown reduces H3K27me3 in the HOX genes cluster and increases HOX gene expression

**Figure 5:**
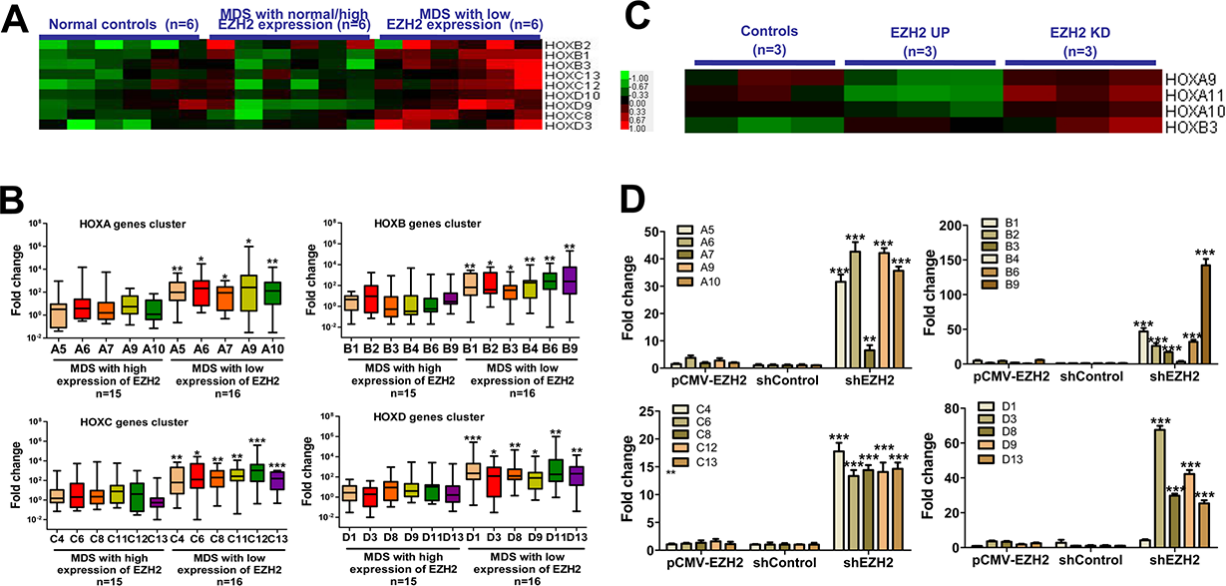
EZH2 knockdown eliminates H3K27me3 marks in the HOX gene cluster and increases HOX gene expression (**A**) GEM analysis of MDS patients revealed higher expression of several HOXB, HOXC, and HOXD genes in patients with low EZH2 expression. (**B**) QRT-PCR revealed increased HOX gene expression in patients with low EZH2 expression. (**C**) GEM analysis of the EZH2 knockdown cells revealed higher expression of HOXA and HOXB genes. (**D**) QRT-PCR confirmed that elevated HOX gene cluster expression was observed in SKM-1 cells with EZH2 knockdown. Statistical significance relative to the control group is indicated: *p < 0.05; **p < 0.01; ***p < 0.001 (two-tailed, Student's *t*-test). Error bars show SEM.

We evaluated whether knockdown of EZH2 resulted in a loss of H3K27me3 in the HOX cluster loci. ChIP-qPCR analysis revealed a reduction in H3K27me3 in the HOXA cluster (HOXA5, A6, A7, A9 and A10), the HOXB cluster (HOXB4, B6, B8, B9 and B13), the HOXC cluster (HOXC4, C6, C11, C12 and C13) and the HOXD cluster (HOXD1, D3, D8, D9 and D13) in SKM-1 cells with EZH2 knockdown compared to control cells (Figure 4F). Thus, EZH2 depletion reduced genome-wide H3K27me3 at the HOX clusters in SKM-1 cells. Additionally, we investigated the association between EZH2 expression and HOX gene expression in primary MDS patients. Gene expression microarray (GEM) analysis revealed high expression of several HOXB, HOXC, and HOXD genes in patients with low EZH2 expression (Figure 5A). qRT-PCR confirmed that HOX cluster gene expression was increased in patients with low EZH2 expression compared to those with high or normal EZH2 expression (Figure 5B). Similarly, GEM analysis of the EZH2 knockdown cells revealed increased HOXA and HOXB gene expression (Figure 5C). qRT-PCR analysis confirmed that HOX cluster gene expression was elevated in SKM-1 cells with EZH2 knockdown (Figure 5D).

### H3K27 hypomethylating agents induce HOX gene cluster expression

**Figure 6:**
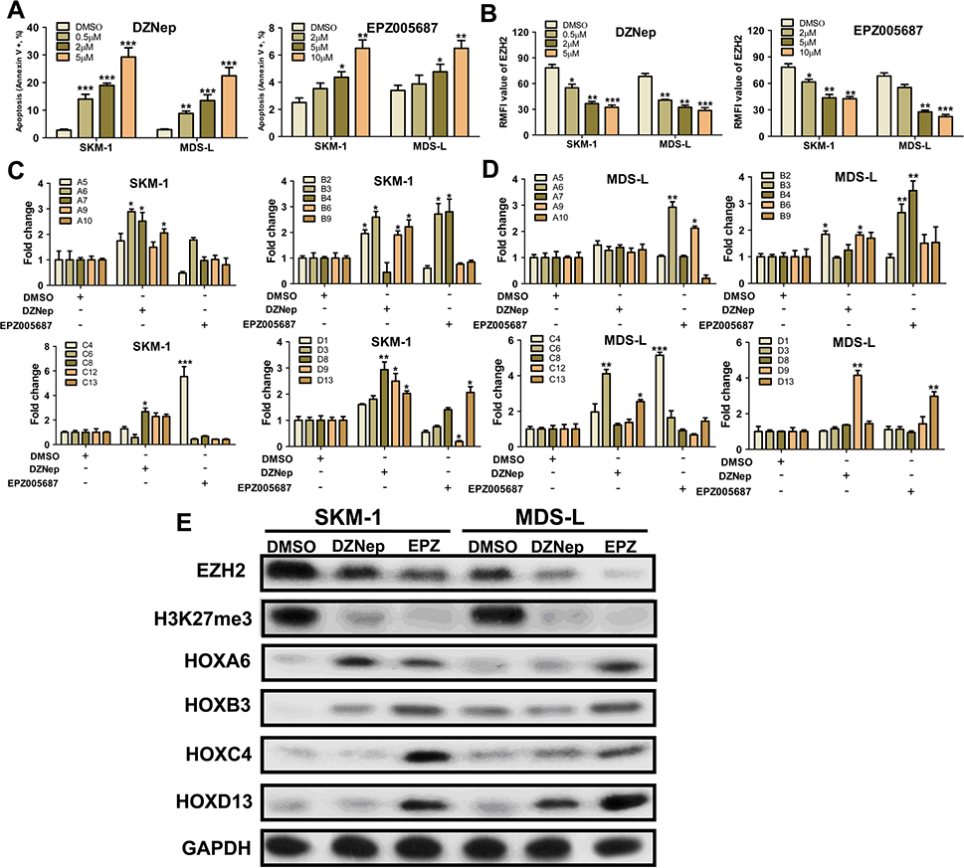
H3K27 hypomethylating agents induce varying degrees of HOX gene cluster expression (**A**) DZNep and EPZ005687 increased cell apoptosis in SKM-1 and MDS-L cells in a dose-dependent manner. (**B**) DZNep and EPZ005687 reduced EZH2 expression in SKM-1 and MDS-L cells. (**C**) and (**D**) Co-treatment with low concentrations (0.5 μM) of DZNep and EPZ005687 increased HOX gene cluster expression in SKM-1 and MDS-L cells. (**E**) Western blot analysis showed that both DZNep and EPZ005687 reduced EZH2 expression and H3K27me3 levels. Statistical significance relative to the control group is indicated: *p < 0.05; **p < 0.01; ***p < 0.001 (two-tailed, Student's *t*-test). Error bars show SEM.

The effect of the H3K27 hypomethylating agents DZNep and EPZ005687 on HOX gene expression was investigated in SKM-1 and MDS-L cells. After treatment (48 h) with DZNep (0.5–5 μM) or EPZ005687 (0.5–5 μM), cell apoptosis in both SKM-1 and MDS-L cells increased in a dose-dependent manner (Figure 6A). DZNep and EPZ005687 also reduced EZH2 expression in these cells (Figure 6B). QRT-PCR results indicated that co-treatment with low concentrations of DZNep (0.5 μM) and EPZ005687 (0.5 μM) increased HOX gene expression in SKM-1 and MDS-L cells (Figure 6C and 6D). Furthermore, western blot analysis showed that both DZNep and EPZ005687 reduced EZH2 expression and H3K27me3 levels (Figure 6E). Increased expression of representative HOX genes such as HOXA6, HOXB3, HOXC4, and HOXD13 was observed after treatment with low concentrations of DZNep and EPZ005687. Together, these results indicate that H3K27 hypomethylating agents might contribute to oncogenic activation of the HOX gene clusters.

## DISCUSSION

Our current data show that the low EZH2 expression observed in high-grade MDS patients is associated with shorter survival and increased AML transformation. Two other studies also found low EZH2 expression in high-grade MDS [22, 23]. However, previous research has also found that EZH2 overexpression is common and is associated with poor prognosis in solid cancers and leukemia [9–15]. EZH2 may therefore act as a tumor suppressor in MDS but as an oncogene in solid cancers and leukemia. The specific genetic environment of MDS cells might account for this difference in the effect of EZH2. An analysis that combined an SNP-array with MC revealed that approximately 30% of MDS patients, especially high-grade patients and those with MDS-derived AML, have a loss of heterozygosity in chromosome 7 [23]. We also detected 7q- abnormalities in many MDS patients (40%) using an SNP array. Because EZH2 CN loss (located in 7q36.1) is common in MDS, EZH2 overexpression may be rare in high-grade patients, while solid cancers and lymphoma are rarely associated with genomic lesions of EZH2. Genomic loss of EZH2 may therefore reduce the expression of EZH2 in MDS.

In addition to low EZH2 expression, we found that EZH2 mutations were also important in MDS pathogenesis. However, the effect of EZH2 mutations on H3K27me3 in MDS remains controversial. Some EZH2 mutations are considered to be inactive because they involve reduced H3K27me3 catalytic activity [19]. Other EZH2 mutations, including the EZH2 Y641 mutant, increase H3K27me3 catalytic activity in MDS and lymphoma [24–27]. Because EZH2 mutations often co-occur with chromosome 7 abnormalities, such as deletion and UPD, active and inactive EZH2 mutations may be secondary changes resulting from EZH2 deletion. Therefore, low EZH2 expression may contribute to MDS pathogenesis more than EZH2 mutations in MDS, which occur with lower frequency. In addition, a recent study revealed that SRSF2 mutations trigger mis-splicing of EZH2 and result in the degradation of EZH2 mRNA in MDS [28]. This genomic EZH2 insufficiency led to attenuated PRC2 function, which may impair the development of hematopoietic cells.

We found that EZP2 knockdown increased proliferation in SKM-1 cells and in a xenograft model, which is consistent with the clinical results. As discussed above and shown in the results, genomic EZH2 deficiency results in low EZH2 expression and genome-wide reductions in H3K27me3. The HOX gene cluster is activated during embryonic development and inactivated in adult cells through increases in the H3K27me3 chromatin mark [29]. Therefore, the loss of EZH2 facilitates tumorigenesis by activating the HOX genes cluster, although inhibition of epigenetic modifiers in solid cancers contributes to re-activation of TSGs and blocks cancer progression. This effect of decreased EZH2 levels on the HOX gene cluster is also reported in other studies [30, 31]. For example, EZH2 knockdown enhanced HOXA gene cluster expression in lymphoma cells. However, these results and our data suggest that the function of the HOX gene cluster differs in different hematopoietic malignancies. EZH2 activation and HOX inhibition predominate in lymphoma, while EZH2 inhibition and HOX gene activation are features of MDS. The biological effects of the EZH2/HOX axis may depend on its specific function in different cancers or in different disease stages within a single cancer type.

Some patients with MDS have benefited from treatments that affect epigenetic modifications. However, frequent relapses that are more difficult to treat are observed in MDS patients receiving epigenetic therapy [6]. In this study, we analyzed HOX gene cluster expression after treatment with H3K27 demethylating agent. Surprisingly, the histone demethylating agent increased HOX gene cluster expression despite increased cell apoptosis. However, accumulation of HOX gene cluster products may increase turbulent cell proliferation, which is not affected by epigenetic agents. The reversible nature of epigenetic changes suggests that reactivation of silenced oncogenes in MDS after epigenetic treatments, rather than the treatments themselves, may result in drug resistance.

In sum, our findings suggest genomic EZH2 loss contributes to epigenetic-dependent overexpression of the HOX genes in MD. But the consequences of altered EZH2 expression are complicated, and caution should be used when choosing treatments that may alter EZH2 levels.

## MATERIALS AND METHODS

### Patients and cells

A total of 97 MDS patients and 52 healthy volunteers were included in this study. Bone marrow mononuclear cells (BMNCs) from the MDS patients were separated with Ficoll-Hypaque gradient centrifugation, and CD34+ cells were isolated by MACS cell sorting according to the manufacturer's protocol. SKM-1 and MDS-L cells were maintained in complete medium (RPMI 1640 supplemented with 10% heat-inactivated fetal bovine serum and 1% penicillin-streptomycin). Additionally, IL-3 (100 U/ml), which is essential for MDS-L cells, was added to their medium. Detailed descriptions of MDS patients are provided in the Supplementary Materials and Methods.

### Flow cytometry analysis of EZH2 protein levels

CD34-APC and EZH2-PE as well as corresponding isotype fluorescence controls were purchased from BD Biosciences (Shanghai, China). In brief, BMNCs were stained with anti-CD34 at room temperature for 20 min. After treatment with a lysing solution and a permeabilizing solution, the cells were stained with anti-EZH2-PE at room temperature for 45 min. The expression of EZH2 in the CD34 blasts was quantified using relative mean fluorescence intensity (RMFI) (the mean fluorescence intensity of the antigen staining divided by the mean fluorescence intensity of the isotype-matched negative control staining).

### Copy-number variation (CNV) and loss of heterogeneity (LOH) detection

DNA from the MDS patients was prepared for hybridization with the Affymetrix CytoScan 750 K array (750,000 probes) according to the manufacturer's protocol. Quantitative real-time PCR (QRT-PCR) was used to validate the SNP results. The detailed protocol is described in the Supplementary Materials and Methods.

### RNA preparation and QRT-PCR

Total RNA was extracted from 10^5^ CD34+ cells using the RNeasy system (Qiagen, Valencia, CA) following the manufacturer's instructions, and the RNA was reversed transcribed into cDNA. PCR reactions for EZH2 and HOX gene clusters were performed using the ABI PRISM 7500 System (Applied Biosystems, CA, US) and SYBR Green Master Mix (Takara, Dalian, China). The relative expression of EZH2 and HOX gene clusters was calculated using 2^−ΔΔCT^. The detailed primer sequences are listed in Supplementary Table 2.

### Lentivirus-mediated cell transfection

pCMV-EZH2 and shEZH2 lentiviruses were purchased from TELEBIO (Shanghai, China). Transfection of SKM-1 cells was performed by mixing virus with cell suspensions followed by centrifugation at 30°C for 90 min with 5 mg/ml polybrene in medium. After viral transduction, colonies of cells with stable expression of EZH2, shEZH2, or a control shRNA were established in the presence of puromycin (10 mg/ml) in medium. The detailed protocol is described in the Supplementary Materials and Methods.

### Measurement of cell proliferation, apoptosis, cell cycle, and colony formation

Measurements of cell proliferation, apoptosis, cell cycle status and colony formation were collected according to a previously published protocol [22]. The detailed protocol is described in the Supplementary Materials and Methods.

### Measurement of cell migration and adhesion capacity

For cell migration, transfected cells were plated onto Transwell Permeable Support inserts with an 8-μm microporous membrane (Corning Costar, US). Culture medium containing recombinant human FGF2 was used as a chemoattractant in the lower compartment. Within 6–8 h, cells that migrated to the bottom membranes in the Transwells were counted in five random 100 × fields. Expression of adhesion biomarkers E-cadherin, VE-cadherin, ICAM, and NCAM on cell surfaces was determined using FCM in SKM-1 cells before and after transfection.

### *In vivo* tumorigenicity assay

Transfected SKM-1 cells (2 × 10^7^) were resuspended in 200 μl of D-Hanks and injected subcutaneously into the forelimbs of four- to six-week old nude mice (Balb/c, female). The animals were sacrificed and tumor tissues were acquired after three weeks. The tumor volume was calculated. Two mice were selected at random for use in each experiment, and each experiment was independently repeated five times. The study protocol was approved by the Committee on the Use of Live Animals in Teaching and Research at the Sixth Hospital affiliated with Shanghai Jiao Tong University.

### H3K27 methylation analysis

H3K27me1 and H3K27me3 analyses were performed using the EpiQuik™ Global Histone H3-K27 Di/Tri-Methylation Assay Kit (Epigentek Group Inc., NY, US) according to the manufacturer's instructions. The detailed protocol is described in the Supplementary Materials and Methods.

### Gene expression microarray (GEM)

A GeneChip PrimeView Human Gene Expression Array (Affymetrix, US) was used for the GEM study. Differential gene expression profiles were identified among the normal controls (*n* = 6), the low EZH2 expression group (*n* = 6), and the high EZH2 expression group (*n* = 6). Similarly, differential gene profiles were analyzed among SKM-1 controls (*n* = 3), SKM-1 cells with EZH2 knockdown (*n* = 3), and SKM-1 cells with EZH2 overexpression (*n* = 3). By combining the GEM data from the MDS patients with the *in vitro* data from the cell lines, EZH2-targeted genes were identified. The detailed protocol is described in the Supplementary Materials and Methods.

### Western blotting

Primary antibodies for EZH2, H3K27me3, H3K27me1, and GAPDH and secondary human anti-rabbit antibody were purchased from Cell Signaling Technology (Danvers, MA, US). HOXA6, HOXB3, HOXC4, and HOXD13 antibodies were purchased from Santa Cruz Biotechnology (Dallas, TX, US). The detailed protocol is described in a previous study [32].

### Chromatin immunoprecipitation (ChIP) and ChIP-on-ChIP analysis

ChIP experiments were performed in CD34+ cells from eight patients with MDS, eight normal controls, and the transfected cell lines using the Magna™ Chromatin Immunoprecipitation system (Millipore, MA, US). Ten million CD34+ cells were pooled from two MDS patients with identical subtypes or two normal controls to obtain a sufficient amount of ChIPed DNA for the NimbleGen whole-genome promoter analysis (Roche NimbleGen, Madison, WI, USA). The detailed protocol is described in the Supplementary Materials and Methods.

### Chromatin immunoprecipitation–quantitative polymerase chain reaction (ChIP–qPCR)

The DNA pool from ChIP, the input control, and the IgG control were used for qPCR. PCR amplification was performed on an ABI 7500 real-time PCR machine (Applied Biosystems, Foster City, CA). The percentage input was determined using the 2^(CtChIP-CtInput)^ × 100% method. Fold enrichment was calculated as follows: %(ChIP/Input)/%(IgG control/Input).

### Statistical analysis

All statistical analyses were performed using SPSS 11.0 software. Two-sample *t*-tests (normal distribution) or non-parametric tests (non-normal distribution) were used to compare two independent samples. Multiple pairwise comparisons were made using a one-way analysis of variance (ANOVA). Pearson correlation analyses was used for the numerical tests. Survival curves were plotted using the Kaplan-Meier method, and the curves were compared using the log-rank test. A *p* < 0.05 was considered statistically significant.

## SUPPLEMENTARY MATERIALS TABLES AND FIGURES


