# Supplementary material for: Genomic loss of EZH2 leads to epigenetic modifications and overexpression of the HOX gene clusters in myelodysplastic syndrome
Source: Oncotarget. 2016 Jan 23;7(7):8119–30. doi: 10.18632/oncotarget.6992 (PMC4884980; doi:10.18632/oncotarget.6992)
Supplement: Supplementary file 1 [file oncotarget-07-8119-s001.pdf]

Genomic loss of EZH2 leads to epigenetic modifications and overexpression of the HOX gene clusters in myelodysplastic syndrome

Supplementary Materials

SUPPLEMENTARY MATERIALS AND METHODS

Patients and cells

MDS was diagnosed in accordance with the minimum diagnostic criteria established by the Conference on MDS (Vienna, 2006) [1].The classification of MDS and prognostic risk scoring were performed according to the WHO criteria and the International Prognostic Scoring System (IPSS) [2, 3]. A total of 97 MDS patients, including 48 low-grade and 49 high-grade patients, were included in this study. The patients who received any treatment, including chemotherapy, epigenetic therapy, etc., were excluded. In addition, 52 healthy volunteers and patients with benign cytopenias were assigned to the normal control group. All subjects provided informed consent in accordance with the Declaration of Helsinki. The research was approved by the ethics committee of the Sixth Hospital affiliated with Shanghai Jiao Tong University. Bone marrow mononuclear cells (BMNCs) from MDS patients were separated with Ficoll-Hypaque gradient centrifugation, and CD34+ cells were isolated by magnetic-activated cell sorting (MACS, Miltenyi, German) according to the manufacturer's protocol. The SKM-1 cells (taken from a patient with MDS-derived AML) were a gift from Prof. Nakagawa (Hyogo Medical Centre) [4].The MDS-L cells (constructed from a patient with RAEB-2) were a gift from Prof. Tohyama (Hamamatsu University School of Medicine) [5]. The cell lines were maintained in complete medium (RPMI 1640 supplemented with 10% heat-inactivated fetal bovine serum, 1% glutamine and 1% sodium pyruvate). Additionally, IL-3 (100 U/ml) was included in MDS-L cell medium.

Copy-number variation (CNV) and loss of heterogeneity (LOH) detection

The DNA from the MDS groups was prepared for hybridization with the Affymetrix CytoScan 750K array (750,000 probes) according to the manufacturer's protocol. A total of 250 ng of isolated DNA from each sample was digested with NspI, ligated, PCR amplified and purified, fragmented, biotin-labeled, and hybridized for a CytoScan 750K Array (Affymetrix, Santa Clara, USA). The data were analyzed using the Nexus Copy Number (version 7.5; Biodiscovery Inc., El Segundo, CA, USA) software program and normalized using the SNP-FASST2 segmentation algorithm. The normalized probe intensity and the allele ratio data were visualized in Nexus v7.5. In addition to the microarray analysis, the TaqMan® Copy Number Assay was used to quantitatively analyze EZH2 copy number. Primers and probes were purchased from Applied Biosystems Inc. The assay was performed according to the manufacturer's instructions. Each replicate was normalized to TERT (a reference gene) to obtain a ΔCt (FAM dye Ct – VIC dye Ct), and an average ΔCt for each sample was calculated. All samples were normalized to a calibrator sample to determine ΔΔCt. The relative quantity (RQ) is $2^{-\Delta\Delta Ct}$, and the copy number is 2 X RQ.

Lentivirus-mediated cell transfection

A 2256-bp CDS of the EZH2 gene was cloned into the Xba I and Not I sites in the pCDH-CMV-MCS-EF1-copGFP vector (purchased from TELEBIO Inc.). Two EZH2-shRNAs were cloned into the Hpa I and Xho I sites in the pLL3.7 vector (purchased from TELEBIO Inc., Shanghai, CN). The detailed sequence of the shRNA for EZH2 is listed in Supplementary Table 2. The construction of the EZH2-expressing and EZH2-knockdown vectors was confirmed with restriction analysis and DNA sequencing. Lentiviral packaging was performed using a four-plasmid system (pCMV-EZH2/ pLL3.7-shEZH2, pRsv-REV, pMDlg-pRRE and pMD2G). After titre determination ($1-3 \times 10^8$ TU/mL), the pCMV-EZH2 or shEZH2 lentivirus was transfected into SKM-1 cells. In brief, 5×10^5 cells/well in a 6-well plate were incubated with the virus and polybrene (6 µg/ml) in a 1 mL volume, followed by centrifugation at 30°C for 90 min with 5 mg/ml polybrene in medium. After viral transduction, cells with stable expression of the EZH2, shEZH2, or control shRNA were established in the presence of puromycine (10 mg/ml) in medium. The stable expression cells were then propagated in complete 1640 medium at 37°C for 1–2 weeks prior to further experimental manipulations. No noticeable loss of GFP expression in the established cultures was observed through fluorescence microscopy or flow cytometry. Overexpression and

silencing efficiency of the EZH2 vectors were evaluated using qRT-PCR. A decrease greater than 70% decrease in BMI1 mRNA expression post-transfection qualified cells for use in the experiment.

Measurement of cell proliferation, apoptosis, cell cycle, and colony formation

Transfected cells were plated in 96-well plates at a density of 5×10^3 cells/well in triplicate. After culture, ten microliters of WST-1 working solution (Keygen, Nanjing, CN) were added to each well, and cells were incubated for 2 h. The absorbance at 450 nm was measured using a microplate reader. The inhibition rate of cell proliferation was calculated as follows: % inhibition rate = each time point ($OD^{treated\ well} - OD^{blank\ well}$) /original time point ($OD^{unreated\ well} - OD^{blank\ well}$). Apoptosis was evaluated by flow cytometry after staining the cells with anti-Annexin V–FITC and PI (Keygen). For cell-cycle analysis, cells were plated in 24-well plates at 2×10^5 cells/well. At 48 h post-transfection, cells were fixed with 70% ice-cold ethanol, washed with PBS, and then resuspended in 1 mL of PBS containing 50 ug/mL PI and 500 U/mL RNase A. Following incubation for 15 min in the dark at room temperature, cells were analyzed with FACS. The percentages of cells in the G1, S, and G2 phases were calculated using Cellquest software. For colony formation, transfected cells were plated in 12-well plates with Methocult H4434 methylcellulose medium containing SCF, GM-CSF, IL-3, and erythropoietin (StemCell Technologies, Hangzhou, CN) at 2×10^3 cells/well in duplicate wells for each condition. After 14 days in a humidified incubator at 37°C, the number of colonies containing at least 30 cells was counted.

H3K27 methylation analysis

Analysis of H3K27me1 and H3K27me3 was performed using EpiQuik™ Global Histone H3-K27 Di/Tri-Methylation Assay Kit (Epigentek Group Inc, NY, US). The assay was carried out according to the manufacturer's instructions. In brief, 1×10^6 transfected cells were lysed with cell lysis buffer, and total histone was extracted. Histone extraction was coated onto assay wells, and antibodies recognizing H3K27me1/3 were added after washing. Finally, HRP conjugated secondary antibody was added and absorbance at 450 nm was measured using a microplate reader after incubation. H3-K27 methylation was calculated as follows: Methylation % = OD (sample-blank)/OD (untreated control-blank) × 100%.

Gene expression microarray (GEM)

A GeneChip PrimeView Human Gene Expression Array (Affymetrix) was used for the GEM study. Signal intensities were acquired with a GeneChip Scanner 3000 7G (Affymetrix) which generated cell intensity files (CEL). Statistical analysis was performed using Partek Genomics Suite software (Partek Inc., St. Louis, MO, USA). A robust multi-array average (RMA) algorithm was used to normalize the data. The false discovery rate (FDR) was less than 0.1, which minimizes false identification of genes. Hierarchical clustering based on the genes and samples was performed with the Cluster 3.0 software. Differential gene expression profiles were identified among the normal controls (n = 6), the low EZH2 expression group (n = 6), and the high EZH2 expression group (n = 6). Similarly, differential gene profiles were analyzed among SKM-1 controls (n = 6), SKM-1 cells with EZH2 knockdown (n = 6), and SKM-1 cells with EZH2 overexpression (n = 3). By combining the GEM data from the MDS patients with the *in vitro* data from the cell lines, EZH2-targeted genes were identified.

Chromatin immunoprecipitation (ChIP) and ChIP-on-ChIP analysis

ChIP experiments were performed in CD34+ cells from eight patients with MDS and eight normal controls and in the transfected cell lines using the Magna™ Chromatin Immunoprecipitation system (Millipore). Ten million CD34+ cells were pooled from two MDS patients with identical subtypes or two normal controls to obtain a sufficient amount of ChIPed DNA for the NimbleGen whole-genome promoter analysis (Roche NimbleGen, Madison, WI, USA). Briefly, 10^7 cells were resuspended in lysis buffer and sonicated to obtain chromatin fragments between 200 bp and 1000 bp. Sonicated chromatin was resuspended in IP buffer and incubated overnight at 4°C with magnetic beads conjugated with either anti-H3K27me3 or anti-EZH2 antibody (Millipore). The immunoprecipitate was washed with lysis buffer, LiCl buffer, and TE buffer, and eluted in elution buffer. DNA was recovered by reversing the crosslinks and purified with a QIAGEN Purification Kit. An unenriched DNA sample was treated in a similar manner to serve as input. Immunoprecipitated DNA was tested for enrichment of control loci using qPCR. For ChIP-on-ChIP analysis, purified ChIP and input DNA was amplified with a WGA amplification kit (Sigma, USA) to obtain sufficient DNA for hybridization, and DNA was then fluorescently labeled using the NimbleGen Dual-Color DNA Labeling Kit according to the manufacturer's protocol (Nimblegen Systems, Inc., Madison, WI, USA). The Cy5-ChIP and Cy3-input labeled DNA samples were co-hybridized to Human CpG Island Plus RefSeq Promoter 3x720K RefSeq Promoter Arrays for 18 h. Microarrays were washed post-hybridization and then scanned using an Axon 4000B microarray scanner with GenePix 6.0. The data were extracted according to standard procedures developed by NimbleGen Systems.

REFERENCES

1. Valent P, Horny HP, Bennett JM, Fonatsch C, Germing U, Greenberg P, Haferlach T, Haase D, Kolb HJ, Krieger O, Loken M, van de Loosdrecht A, Ogata K, et al. Definitions and standards in the diagnosis and treatment of the myelodysplastic syndromes: consensus statements and report from a working conference. Leuk Res. 2007; 31:727–736.
2. Vardiman JW, Harris NL, Brunning RD. The World Health Organization (WHO) classification of the myeloid neoplasms. Blood. 2002; 100:2292–302.
3. Greenberg P, Cox C, LeBeau MM, Fenaux P, Morel P, Sanz G, Sanz M, Vallespi T, Hamblin T, Oscier D, Ohyashiki K, Toyama K, Aul C, et al. International scoring system for evaluating prognosis in myelodysplastic syndromes. Blood. 1997; 89:2079–2088.
4. Tohyama K, Tohyama Y, Nakayama T, Ueda T, Nakamura T, Yoshida Y. A novel factor-dependent human myelodysplastic cell line, MDS92, contains haemopoietic cells of several lineages. Br J Haematol. 1995; 91:795–799.
5. Nakagawa T, Matozaki S. The SKM-1 leukemic cell line established from a patient with progression to myelomonocytic leukemia in myelodysplastic syndrome (MDS)-contribution to better understanding of MDS. Leuk Lymphoma. 1995; 17:335–339.

Supplementary Table S1: Clinical characteristics of 97 patients with MDS

Characteristic	Value
**Age, median (range)**	58 (17–85)
**Male:female**	57:40
**Diagnosis, *n* (%)**	97
RA	12 (12.4)
RARS	3 (3.1)
RCMD	25 (25.8)
RAEB-1	23 (23.7)
RAEB-2	26 (26.8)
MDS-U	8 (8.2)
**Karyotype**	
normal	61 (62.9)
−5/5q−	6 (6.2)
−7/7q−	7 (5.2)
+8	9 (9.3)
−20/20q−	6 (6.2)
Complex	4 (4.1)
others	4 (4.1)
**Blast percentage in marrow, %**	
< 2%	32 (33.0)
2–5%	16 (16.5)
5–10%	23 (23.7)
≥ 10%	26 (26.8)
**Myelofibrosis**	
Negative	32 (33.0)
Mild	41 (42.3)
Moderate	15 (15.5)
Severe	9 (9.3)
**IPSS**	
Lower risk	44 (45.4)
Higher risk	53 (54.6)
**Marrow cellularity**	
Hypo	15 (15.5)
Hyper	82 (84.5)
**EZH2 mutations**	
Yes	5 (5.2)
No	92 (94.8)

Supplementary Table S2: Primer sequences

QRT-PCR		Tm
**EZH2**	AATCAGAGTACATGCGACTGAGA	60.9
	GCTGTATCCTTCGCTGTTTCC	61.0
**GAPDH**	GGAGCGAGATCCCTCCAAAAT	61.6
	GGCTGTTGTCATACTTCTCATGG	60.9
**HOXA5**	AACTCATTTTGCGGTCGCTAT	60.4
	TCCCTGAATTGCTCGCTCAC	62.2
**HOXA6**	TCCCGGACAAGACGTACAC	61.0
	CGCCACTGAGGTCCTTATCA	61.0
**HOXA7**	TCGTATTATGTGAACGCGCTT	60.2
	CAAGAAGTCGGCTCGGCATT	62.8
**HOXA9**	TACGTGGACTCGTTCCTGCT	62.7
	CGTCGCCTTGGACTGGAAG	62.7
**HOXA10**	CTCGCCCATAGACCTGTGG	61.5
	GTTCTGCGCGAAAGAGCAC	62.0
**HOXB1**	AGGAGACGGAGGCTATTTTCA	60.3
	GTCTGCTCGTTCCCATAAGGG	62.2
**HOXB2**	CGCCAGGATTCACCTTTCCTT	62.4
	CCCTGTAGGCTAGGGGAGAG	62.1
**HOXB3**	CCAGTGCCACTAGCAACAG	60.4
	CGTTTGCCTCGACTCTTTCATC	61.5
**HOXB4**	CGTGAGCACGGTAAACCCC	63.0
	CGAGCGGATCTTGGTGTTG	60.8
**HOXB6**	TCCTATTTCGTGAACTCCACCT	60.5
	GCGGGGTAATGTCTCAGCG	62.8
**HOXB9**	CCATTTCTGGGACGCTTAGCA	61.3
	CGTGAGCACGGTAAACCCC	60.2
**HOXC4**	GAGCGCCAGTATAGCTGCAC	63.0
	GCGACTGTGATTTCTCGGGG	62.9
**HOXC6**	ACAGACCTCAATCGCTCAGGA	62.7
	AGGGGTAAATCTGGATACTGGC	61.0
**HOXC8**	TCCGCCAACACTAACAGTAGC	62.1
	AGCGTGGGGTCTCATCCAT	62.7

**HOXC12**	ATGGGCGAGCATAATCTCCTG	61.7
	CGTGGGTAGGACAGCGAAG	62.1
**HOXC13**	GCCGTCTATACGGACATCCC	61.6
	GGTAGGCGCAAGGCTTCTG	63.0
**HOXD1**	CGGGTCTCACGTCCACTAC	61.4
	GATGCGGTCTGGAAAGCAC	61.1
**HOXD3**	CGGCAACTTCGTCGAGTCC	63.0
	ATGAGGGTCGCAAGGTCCA	62.9
**HOXD8**	AGAAGAATCGAGGTTTCCCACG	62.0
	TCCTTTTTCGTTTCCCCGTCC	62.6
**HOXD9**	GGACTCGCTTATAGGCCATGA	61.1
	GCAAAACTACACGAGGCGAA	60.9
**HOXD13**	CTTCGGCAACGGCTACTACAG	62.8
	TGACACGTCCATGTACTTCTCC	61.4
**shEZH2-1**	TGCTGTCCAAATGCTGGTAACACTGTGTTTTGGCCACTGACT-GACACAGTGTTCAGCATTTGGA	
	CCTGTCCAAATGCTGAACACTGTGTCAGTCAGTGGCCAAAA-CACAGTGTTACCAGCATTTGGAC	
**shEZH2-2**	TGCTGTTCAGTCCCTGCTTCCCTATCGTTTTGGCCACTGACT-GACGATAGGGACAGGGACTGAA	
	CCTGTTCAGTCCCTGTCCCTATCGTCAGTCAGTGGCCAAAAC-GATAGGGAAGCAGGGACTGAAC	

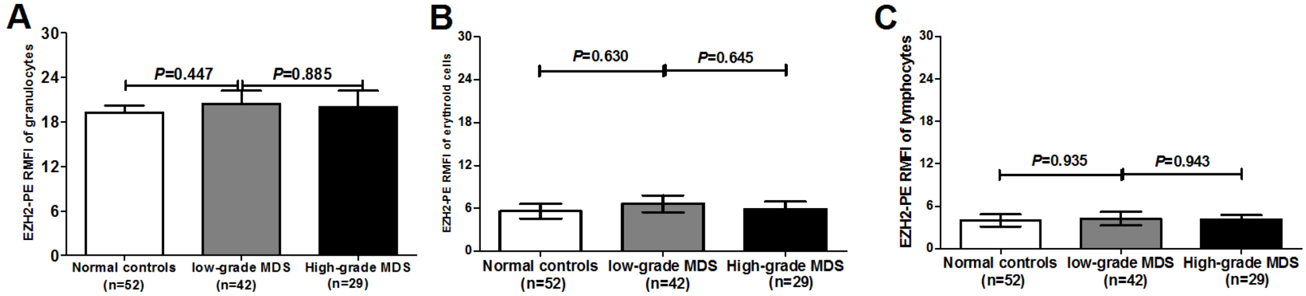

Supplementary Figure S1: EZH2 protein levels in granulocytes, lymphocytes, and erythroid cells. (**A**) In granulocytes, there were no differences in EZH2 levels among the three groups. (**B** and **C**) Lymphocytes and erythroblasts did not express EZH2. *Error bars show SEM.*

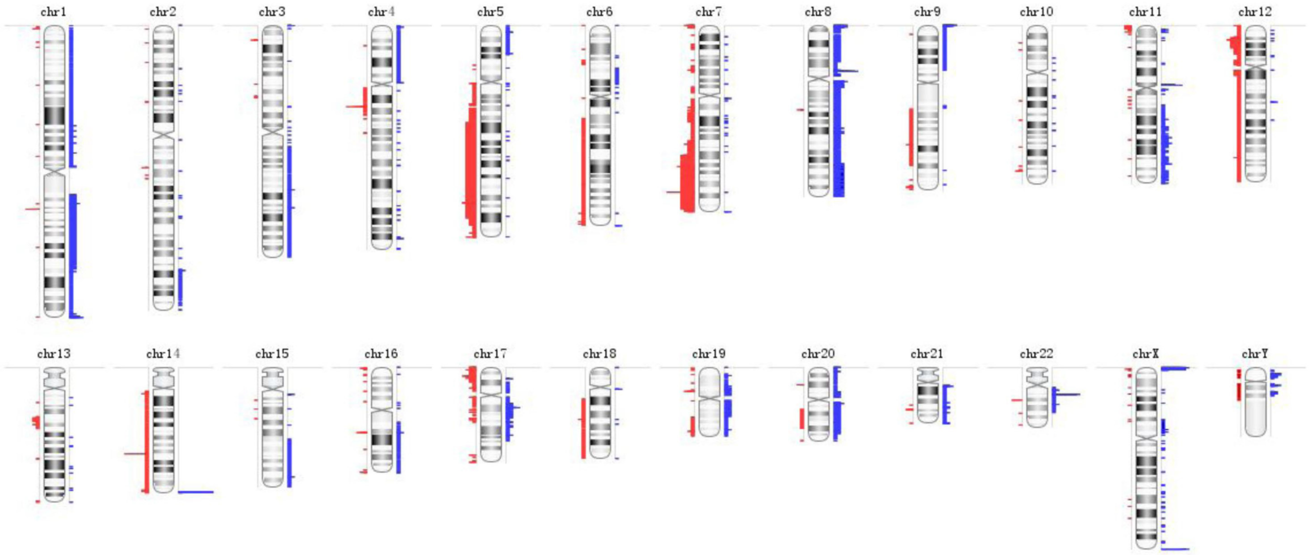

Supplementary Figure S2: Distribution of copy number variations (CNVs) in MDS patients. An SNP array showed that copy number (CN) gain in the chr8 region and CN loss in the chr 7 and chr 5 regions were the most frequent cytogenetic events. CN loss and gain are shown with red and blue lines, respectively.

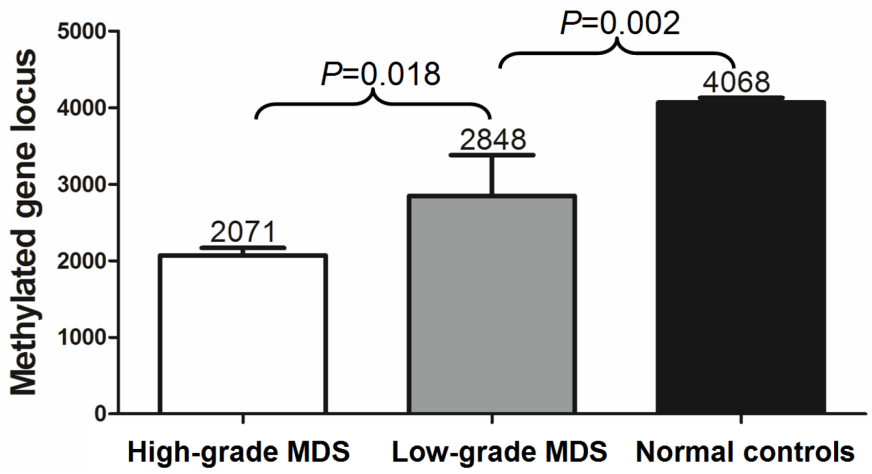

Supplementary Figure S3: ChIP-on-ChIP analysis in MDS patients and normal controls. ChIP-on-ChIP results revealed a reduction in genome-wide H3K27me3 in the MDS group, especially in the high-grade MDS group as compared to the normal controls. *Error bars throughout represent the SEM.*

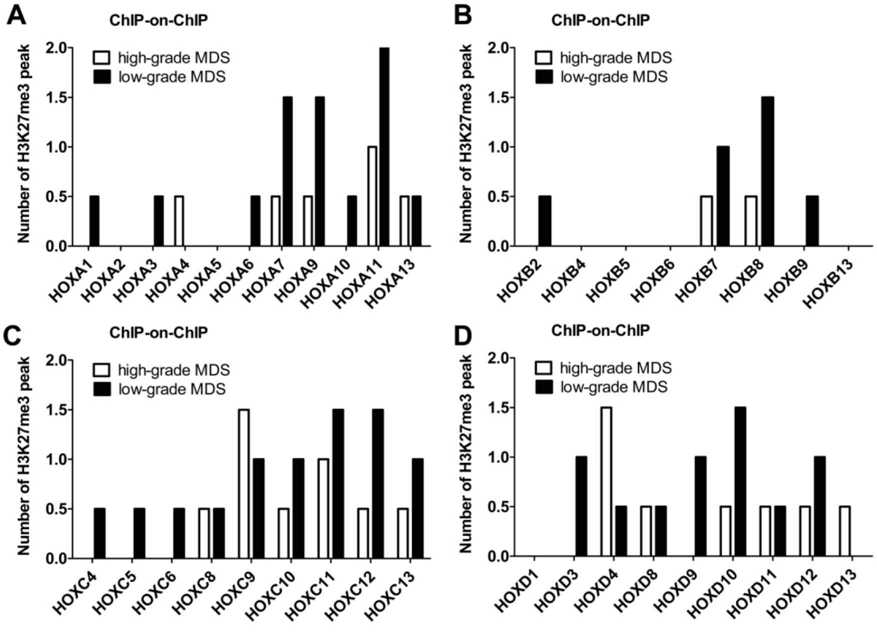

Supplementary Figure S4: Comparison analysis of ChIP-on-ChIP in the low-grade and the high-grade MDS groups. The high-grade MDS group exhibited reduced H3K27me3 compared to the low-grade MDS group in the HOXA (**A**), HOXB (**B**), HOXC (**C**) and HOXD (**D**) cluster loci.
